# Adoptive transfer of dendritic cells modulates immunogenesis and tolerogenesis in a neonatal model of murine cutaneous leishmaniasis

**DOI:** 10.1186/1475-9292-4-2

**Published:** 2005-01-25

**Authors:** Loida V Ponce, José Corado, Nilka L Díaz, Felix J Tapia

**Affiliations:** 1 Laboratorio de Biología Molecular, Instituto de Biomedicina, Universidad de Central Venezuela, Apartado 4043, Caracas 1010A, Venezuela; 2 Departamento de Ciencias Fisiológicas, Universidad de Carabobo, Valencia, Venezuela

## Abstract

We evaluated the adoptive transfer of DCs on *Leishmania (L.) mexicana*-infected neonatal BALB/c mice. DCs were isolated and purified from the spleens of the following donor groups: a) Adult BALB/c mice infected during adulthood with *L. (L) mexicana*; b) Adult BALB/c mice infected during neonatal life; c) Healthy neonatal BALB/c mice; d) Healthy adult BALB/c mice. A neonatal model of infection, generated after inoculation with 5 × 10^5 ^promastigotes of *L. (L) mexicana*, was used as the infection control group. Sixteen hours after intraperitoneal transfer of DCs (1 × 10^3^, 1 × 10^5^, or 1 × 10^6 ^cells/ml), neonatal recipient BALB/c mice were infected. The adoptive transfer of DCs diminished disease progression in neonatal mice. This reduction depends on the quantity and provenance of transferred DCs, since the effect was more evident with high numbers of DCs from adult mice infected during adulthood and healthy neonatal mice. Protection was significantly reduced in animals receiving DCs from healthy adult mice but it was absent in mice receiving DCs from adult mice infected during neonatal life. These results suggest that genetic susceptibility to *Leishmania *infection can be modified during neonatal life, and that the period of life when antigens are encountered is crucial in influencing the capacity of DCs to induce resistance or tolerance.

## Background

Medawar et al. [1] showed almost half a century ago that rodents injected at birth with splenocytes from genetically different donors could accept transplants from that donor as an adult. These milestone experiments guided the notion that the introduction of antigens during neonatal life leads to tolerance and that the immune system functions by making a distinction between self and nonself. For some years, Matzinger et al. have persevered on the hypothesis that tolerance is not an intrinsic property of the newborn immune system [2,3]. For example, many studies have shown that neonatal exposure to antigen may prime T cells and induce both Th1 and Th2 cells [4-7]. Moreover, Adkins et al. have demonstrated that although neonates develop compartmentally distinct primary responses to antigen immunization (mixed Th1/Th2 in lymph nodes and Th2 in spleen), after rechallenge the elicited secondary response is always of the Th2 type [7,8]. They have also proved that even in the lymph nodes, the Th2 function persists for a prolonged period after a single immunization, and that animals initially immunized as neonates are impaired in their capacity to develop the expected Th1 memory effector function observed in adults [9]. The biased immunogenic neonatal immunity may be attributable to factors associated with antigen presentation such as type of antigen-presenting cell, accompanying adjuvant and the nature, concentration and in vivo availability of the antigen [5,10-13]. Resting T cells need two signals to be activated; signal 1 from TCR binding to MHC/peptide and signal 2 (co-stimulation) from a professional phagocyte, such as a dendritic cell or a macrophage. Tolerance is associated to a lack of co-stimulation that usually occurs when antigen is encounter by a non-professional phagocyte, or by professional phagocytes in a non-APC tissue (lymphoid tissue, skin, etc)[14]. In this study, we have evaluated the effect of adoptive transfer of DCs from adult and neonatal mice infected with *L. (L.) mexicana*, and from healthy adult and neonatal mice. As in the *L. major *mouse model, we have shown that infection with *L. (L.) mexicana *strain MHOM/BZ/82/BEL21, generates a Th1 response associated to protective immunity in C57BL/6 mice, and a Th2 response related to non-healing disease in BALB/6 mice [15].

Leishmaniasis is an excellent model to study the extremes of host/parasite relationships, particularly the diversity of the immune response associated to the genetic background of the host. In addition, mice can reproduce the distinct clinical forms observed in humans [16,17]. These models have been particularly important to show that skin-derived DCs including Langerhans cells play an important role in cutaneous leishmaniasis, where they can transport *Leishmania *antigens to the lymph nodes and induce specific immune responses [18-24]. Moll et al. have also shown that Langerhans cells may act as reservoirs sustaining parasite-specific stimulation of T memory cells, thus protecting animals from reinfection [25].

## Results and Discussion

### Establishment of a *L. (L.) mexicana *infection model in neonatal BALB/c mice

**Figure 1:**
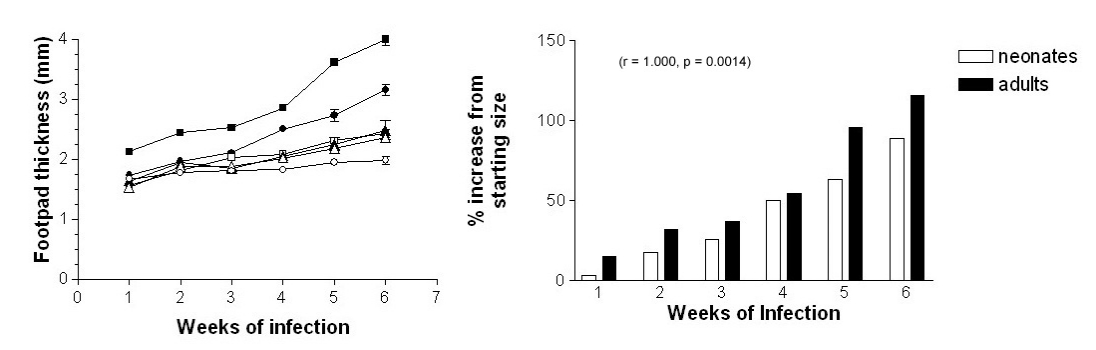
Progression of *L. (L.) mexicana *infection in neonatal BALB/c mice. A. Footpad thickness of adult mice infected with 1 × 10^6 ^promastigotes (■), non-infected mice (○), neonatal mice infected with 5 × 10^4 ^promastigotes (△), neonatal mice infected with 1 × 10^5 ^promastigotes (▲), neonatal mice infected with 2 × 10^5 ^promastigotes (□) and neonatal mice infected with 5 × 10^5 ^promastigotes (●). B. Percentage increase from the starting footpad thickness in both neonatal (□) and (■) adult BALB/c mice infected with 5 × 10^5 ^and 1 × 10^6 ^promastigotes, respectively.

The progress of *L. (L.) mexicana *infection in neonatal BALB/c mice, after the inoculation with 5 × 10^4^, 1 × 10^5^, 2 × 10^5 ^or 5 × 10^5 ^promastigotes was determined by measuring the footpad thickness during 6 weeks. All 4 experimental groups developed lesions. Mice that received 1 × 10^5^, 2 × 10^5 ^and 5 × 10^5 ^promastigotes respectively, showed a significant increase (p ≤ 0.05) on footpad thickness starting from the second week, reaching a maximal value on the sixth week of evaluation (Fig. 1A). This increase in footpad thickness was much greater (p ≤ 0.05) in the group inoculated with 5 × 10^5 ^promastigotes, with lesions appearing from the first week (Fig. 1A). Moreover, this experimental group presented a similar evolution to that observed in *L. (L.) mexicana*-infected adult BALB/c mice inoculated with 1 × 10^6 ^promastigotes (Fig. 1B). The statistical analysis using a Wilcoxon matched-pairs signed-ranks test of the percentage increase from the starting footpad thickness in both neonatal and adult BALB/c mice infected with 5 × 10^5 ^and 1 × 10^6 ^promastigotes, respectively, showed a significant (p ≤ 0.05) two-tailed value and a very significant Spearman correlation (r = 1.000, p = 0.0014). The starting footpad thickness in neonatal and adult BALB/c mice was 1.67 mm and 1.85 mm, respectively.

We used 5 × 10^5 ^promastigotes as the optimal concentration for *L. (L.) mexicana *infection in all the subsequent experiments including the infection control group. This neonatal murine model of *L. (L.) mexicana *infection used half the numbers of promastigotes previously described to infect adult BALB/c mice [16]. A significant Spearman correlation attested that our neonatal model was comparable to the adult model of infection. Although infected neonatal mice have a statistically similar clinical outcome that infected adult mice, we ignore whether these mice have similar level of infection and therefore similar concentrations of antigens carried over by the transferred DCs, however, looking at the present results one can speculate that DCs from mice infected during neonatal life induced tolerance probably due to a high parasite burden, and not a lack of adjuvancity since DCs from healthy neonatal mice were able to partially protect against *Leishmania *infection. Other studies have shown a similar pattern of Th2-biased immune response in other models of neonatal infection [7,11]. We also observed that even after the inoculation of considerable numbers of parasites, neonatal mice differed significantly from adult mice in their percentage increment from the starting footpad thickness, suggesting a functional impairment of the primary immune response. This may be explained, first by the fact that in BALB/c mice carry a point mutation in the Nramp1 (natural-resistance-associated macrophage protein) gene that allows the mRNA degradation of macrophage activation genes, increasing susceptibility to *Leishmania *infection [26]. Susceptibility associated with the dominant expression of the costimulatory molecule CD86 (B7-2) and the subsequent generation of the Th2-mediated response [27-31]. Second, the proof that murine naïve neonatal T cells, unlike adult T cells, express a Th2 phenotype and are highly deficient in Th1 functions [32,33].

### Morphological and immunophenotypic characterization of murine splenic dendritic cells

**Figure 2:**
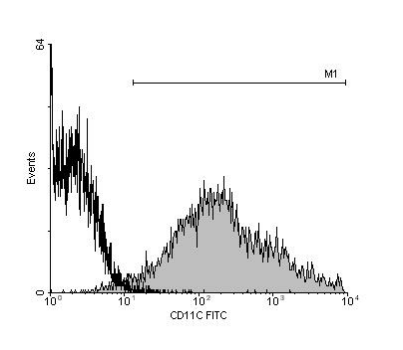
Frequency distributions of purified dendritic cells labelled CD11c-FITC showing 97.17% purity (right), and FITC-isotype control (IgG1) (left). The information shown is from a single cell isolation procedure, representative of various separate experiments.

DCs obtained by our purification method showed characteristic dendritic cell morphology, and a 97% purity as determined by CD11c immunostaining and flow cytometry. A minor fraction of about 3.5 % expressed CD3 and NK1.1 (Fig. 2). The expression of CD11c, MHC-II and CD86 molecules was detected by immunocytochemistry, thus demonstrating that these cells showed characteristics of functionally mature DCs.

Splenic DCs were isolated for our adoptive transfer experiments since they are mobile antigen-presenting cells that migrate to peripheral lymph organs where they stimulate naive T cells, thus initiating primary T cell responses [34-36]. Further, splenic DCs have been isolated by standardized procedures based on the high expression of CD11c and the lack of CD205 [37].

### Progression of the infection in neonatal recipient BALB/c mice after adoptive transfer of dendritic cells from the distinct experimental groups

**Figure 3:**
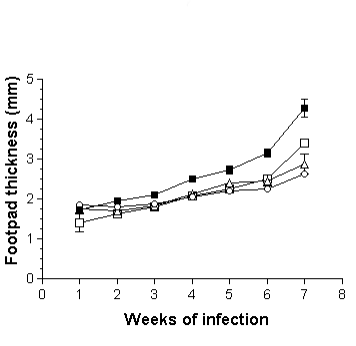
Progression of infection in neonatal recipient BALB/c mice after adoptive transfer of DCs from adult BALB/c mice infected during adulthood with *L. (L) mexicana*. Footpad thickness of neonatal mice infected with 5 × 10^5 ^promastigotes (■); neonatal mice transferred with 1 × 10^6 ^(○;), 1 × 10^5 ^(△) and 1 × 10^3 ^(□) DCs and subsequently infected with 5 × 10^5 ^promastigotes.

**Figure 4:**
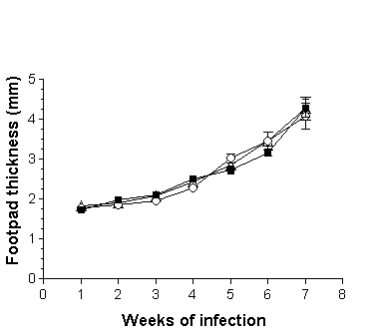
Progression of the infection in neonatal recipient BALB/c mice after adoptive transfer of DCs from adult BALB/c mice infected during neonatal life with *L. (L) mexicana*. Footpad thickness of neonatal mice infected with 5 × 10^5 ^promastigotes (■); neonatal mice transferred with 1 × 10^6 ^(○) and 1 × 10^5 ^(△) DCs and subsequently infected with 5 × 10^5 ^promastigotes.

**Figure 5:**
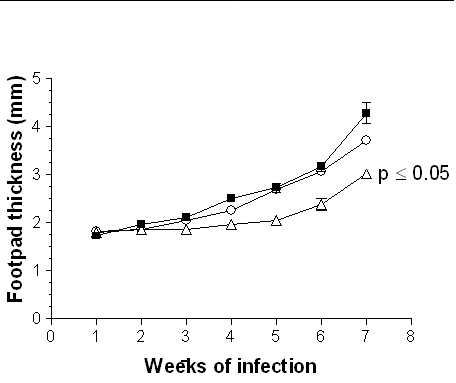
Progression of the infection in neonatal recipient BALB/c mice after adoptive transfer of DCs from healthy adult BALB/c mice. Footpad thickness of neonatal mice infected with 5 × 10^5 ^promastigotes (■); neonatal mice transferred with 1 × 10^5 ^(○) and 1 × 10^6 ^(△) DCs and subsequently infected with 5 × 10^5 ^promastigotes.

**Figure 6:**
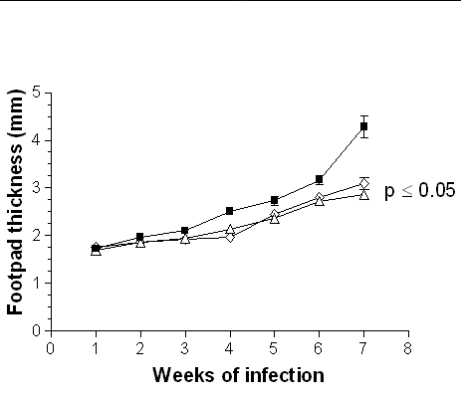
Progression of the infection in neonatal recipient BALB/c mice after adoptive transfer of DCs from healthy neonatal BALB/c mice. Footpad thickness of neonatal mice infected with 5 × 10^5 ^promastigotes (■); neonatal mice transferred with 1 × 10^5 ^(△) and 1 × 10^3 ^(◇) DCs and subsequently infected with 5 × 10^5 ^promastigotes.

The adoptive transfer of 1 × 10^3^, 1 × 10^5 ^or 1 × 10^6 ^DCs from adult BALB/c mice infected during adulthood with *L. (L) mexicana *on neonatal recipient mice modified the course of infection, showing a delayed lesion growth from the second week onward (Fig. 3) as compared with the infection control group. This reduction in footpad thickness was dependent of DC numbers, since at the highest concentration of 1 × 10^6^, lesions were smaller than those observed with 1 × 10^3 ^and 1 × 10^5 ^DCs from the fifth week onward (Fig. 3). At the seventh week of infection, lesion size decreased by 40% after the adoptive transfer of 1 × 10^6 ^DCs, whereas in animals inoculated with 1 × 10^5 ^and 1 × 10^3 ^DCs the decrease was of 33% and 22%, respectively. In contrast, the adoptive transfer of 1 × 10^5^or 1 × 10^6 ^DCs from adult BALB/c mice infected during neonatal life with *L. (L) mexicana *fail to modify the course of infection of neonatal recipient BALB/c mice as compared with infection control animals (Fig. 4). However, those mice receiving 1 × 10^6 ^DCs showed a significant reduction in lesion growth (p ≤ 0.05) on weeks 2, 3 and 4. This effect disappeared from the fifth week onwards (Fig. 4). Moreover, the adoptive transfer of 1 × 10^5 ^or 1 × 10^6 ^DCs from healthy adult BALB/c mice modified the course of infection in neonatal recipient mice, showing a delayed and significant decrease (p ≤ 0.05) in lesion growth from the second week of infection (Fig. 5). This reduction in footpad thickness was dependent on DC numbers, since at 1 × 10^6 ^lesions were significantly (p ≤ 0.05) smaller than those observed in mice transferred with 1 × 10^5 ^DCs, which also initiated their lesions on the third week (Fig. 5). At the seventh week of infection, lesion size decreased by 30% after the adoptive transfer of 1 × 10^6 ^DCs and by 10% in mice receiving 1 × 10^5 ^DCs. similarly, the adoptive transfer of 1 × 10^3 ^or 1 × 10^5 ^DCs from healthy neonatal BALB/c mice modified the course of infection of neonatal recipient mice, showing a delayed and significant decrease (p ≤ 0.05) in lesion growth from the second week of infection. This reduction in footpad thickness was very similar in both tested concentrations (Fig. 6). At the seventh week of infection, lesion size decreased by 35% in both groups.

**Figure 7:**
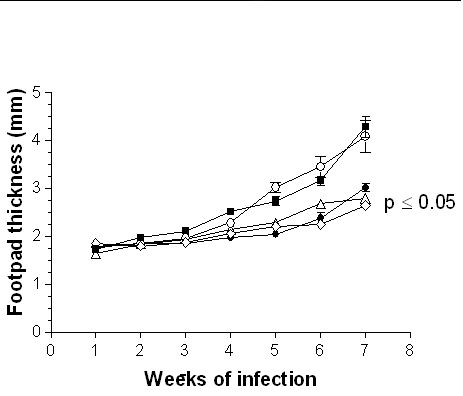
Progression of the infection in neonatal recipient BALB/c mice after the adoptive transfer of DCs from the different experimental groups. Footpad thickness of neonatal mice infected with 5 × 10^5 ^promastigotes (■); neonatal mice transferred with 1 × 10^6 ^DCs from adult BALB/c mice infected during adulthood (◇), adult BALB/c mice infected during neonatal life (○), healthy adult BALB/c mice (●) and 1 × 10^5 ^CDs from healthy neonatal BALB/c mice (△) and subsequently infected with 5 × 10^5 ^promastigotes.

Disease progression was substantially decreased after transferring cells from adult BALB/c mice infected during adulthood with *L. (L) mexicana*, healthy adult BALB/c mice and healthy neonatal BALB/c mice. The reduction in these 3 groups was statistically significant (p ≤ 0.05) as compared with the infection control group. This reduction in footpad thickness was absent or considerably diminished in mice receiving DCs from adult BALB/c mice infected during neonatal life with *L. (L) mexicana *(Fig. 7).

Our results showed that the preceding intraperitoneal adoptive transfer of DCs diminished the progression of *L. (L.) mexicana *infection in neonatal BALB/c recipient mice. These results contrast with those of Moll and Berberich [38] showing that only intravenous administration of antigen-pulsed Langerhans cells, but not intradermal or intraperitoneal inoculation, induced resistance against *Leishmania *infection. In this study, the observed protection depends on the quantity and provenance of the transferred DCs, since the effect was more evident with high cellular numbers of DCs from adult BALB/c mice infected during adulthood and healthy neonatal mice, where lesions were about 40% smaller than in the infection control group. DCs from these two groups have the intrinsic capacity to induce protective or resistant immune responses very early in life. That neonatal DCs appear to be more protective, on a per cell basis, than adults DCs is a very striking result since only Dadaglio et al.[39] have shown that neonatal DCs are as effective as adult DCs in expressing MHC and costimulatory molecules; taking-up, processing and presenting antigens to T cells inducing CTL responses in vivo. Others have shown that neonatal DCs are not fully functional [40,41]. Also, animals receiving DCs from healthy adult mice showed a slightly but significantly reduced protection from that observed with DCs from adult mice infected during adulthood and healthy neonatal mice. Various studies have shown that epidermal DCs in aged skin are reduced significantly compared with young skin in mice and humans [42-48]. This cellular reduction may be the consequence of a decreased production in the bone marrow of DC progenitors or alternatively, these stem cells may be less responsive to cytokine and chemokine signals required for their homing to the skin [49-51]. Our results favor the latter hypothesis, since the same numbers of transferred DCs from healthy neonatal or adult mice induced a somewhat different disease outcome. More notable was the observed absence of a protective effect in mice receiving DCs from adult BALB/c mice infected with *L. (L) mexicana *during neonatal life. This result confirmed recent studies by Adkins et al. showing that animals initially immunized as neonates are unable to develop the expected Th1 memory effector function observed in adults [9]. These investigators proposed that in neonates, the spleen is the primary site of tolerance induction to self-antigens whereas the lymph nodes are the sites of immune responsiveness to foreign antigens. The initial and transitory protection observed at the greatest concentration of DCs from adult mice infected during neonatal period, suggests impairment in their accessory functions specifically in those associated with signal 2 and signal 3. Signal 2 comprises co-stimulatory factors essential for the clonal expansion of T cells and signal 3 involves in situ properties of DCs such as tissue interaction and migration where cytokines, chemokines and extracellular matrix components are crucial [36].

## Conclusions

Our results show that tolerizing DCs from animals initially immunized as neonates play a key role in the attenuation of Th1 responses. The present results may have a considerable epidemiological impact on leishmaniasis, where infection at early stages of life may impose a tolerogenic state that favors the development of visceral or diffuse cutaneous leishmaniasis, both characterized by Th2-type responses.

In this study, we have shown that intraperitoneal adoptive transfer of splenic DCs is able to surpass the genetic bias of the mice, allowing the development of an immune response that modifies the progression of *L. (L.) mexicana *infection.

## Methods

### Animals

Adult (6 weeks) and neonatal (about 24 hour newborn) female BALB/c mice (Taconic, Germantown, NY, U.S.A.) were raised in the Animal House of the Instituto de Biomedicina, under appropriate conditions of temperature, water and feeding.

### Specific Antibodies

The following rat monoclonal antibodies were used to isolate and characterize dendritic cells: CD19 (B cells, clone IBL-2), MOMA-2 (Macrophages/Monocytes), CD45R (B and NK cells; clone RA3-6B2), CD3 (T cells; clone KT3), CD11c (dendritic cells and other leukocytes, clone N418), CD19 (clone 6D5) conjugated to phycoeritrine (PE), NK1.1 (clone PK136) conjugated to PE, Macrophages-Monocytes (MOMA-2) conjugated to fluorescein isothiocyanate (FITC), CD3 (clone KT3) conjugated to FITC. All were purchased from Serotec Ltd. (Oxford, United Kingdom) except CD205 (dendritic cells, clone NLDC-145, DEC205) donated by Georg Kraal, Vrije Universiteit, Amsterdam, The Netherlands; I-A^d ^(MHC-II, clone AMS-32.1) and CD86 (B7.2, clone GL1) purchased from BD Pharmigen (San Diego, USA).

### Parasite culture and isolation of *L. (L.) mexicana *promastigotes

Amastigotes of *Leishmania (Leishmania) mexicana *(MHOM/BZ/82/BEL21) were extracted from footpad nodules of hamsters infected a month earlier with 1 × 10^6 ^amastigotes. The nodules were aseptically dissected out and washed in phosphate-buffered saline (PBS, pH 7.4) with 100 U/ml penicillin and 100 μg/ml streptomycin, and finely cut and ground in a Petri dish containing cold PBS. The suspension was filtered through a sterile sieve to remove large debris. These parasites were cultured on blood agar base (Sigma-Aldrich, St. Louis, U.S.A.) at room temperature for 7 days (the stationary growth phase) to obtain infective promastigotes. For an enriched population of parasites, free of erythrocytes and cellular debris, 100 μl of that sample were cultured in 2 ml Schneider's insect cell culture medium (Sigma-Aldrich, St. Louis, U.S.A.) for one week at room temperature. Promastigotes were isolated after 3 washes with sterile PBS and centrifugation at 1000 g at 4°C for 15 min. Pellets were resuspended in 1 ml of sterile PBS. Viable parasites were counted by trypan blue exclusion. Parasite concentration was adjusted to 5 × 10^4^, 1 × 10^5^, 2 × 10^5 ^and 5 × 10^5 ^per μl to be used in the different experimental groups.

### Experimental infection with promastigotes of *L. (L.) mexicana*

A similar pattern of *L. (L.) mexicana *infection to that established in adult mice [52] was determined in neonatal BALB/c mice. Neonatal BALB/c mice (n = 12) were inoculated subcutaneouslly into the left hind footpad with 5 × 10^4^, 1 × 10^5^, 2 × 10^5^, or 5 × 10^5 ^promastigotes suspended in 10 μl sterile PBS, applied with a tuberculin syringe (29-gauge needle) connected to a stepper repetitive pipette (Tridak, Danbury, U.S.A.). For comparison, adult BALB/c mice were infected the standardized optimal parasite load of 1 × 10^6 ^promastigotes of *L. (L.) mexicana *[52]. The course of infection was evaluated weekly for 6 weeks, measuring the experimental left footpad using a dial gauge caliper (Mituyoto N° 7300, U.S.A.).

### Isolation and purification of dendritic cells

DCs from adult and neonatal BALB/c mice were isolated from the spleen. Under sterile conditions, spleens were minced on a metallic mesh with RPMI-1640 (Life Technologies, Rockville, U.S.A.) supplemented with 10% of decomplemented fetal bovine serum (FBS), 2 mM L-glutamine, 10 mM HEPES, 1 mM sodium pyruvate, 50 μM 2-mercaptoethanol and 100 U/ml penicillin (complete RPMI-10). The cell suspension was filtered on a nylon sieve and transferred to 15 ml centrifuge tubes (Corning Life Sciences, Acton, U.S.A.) and spun at 250 g at 4°C for 10 min. Viable cells were counted by trypan blue exclusion. Cell concentration was adjusted to 1 × 10^7 ^cells/ml in complete RPMI-10 and 8 ml plated in tissue culture flasks. The flaks were incubated for 2 hr at 37°C in a 5% CO_2 _incubator (NuAire, Inc., Plymouth, U.S.A.), allowing DCs to adhere. Non adherent cells were carefully removed and placed in sterile 50 ml centrifuge tubes and spun at 250 g, 4°C for 10 min. Adherent cells were covered with 10 ml complete RPMI-10 and incubated as before for 16–18 hours, allowing DCs to detach. After gently washing the surface of the flasks with a plugged Pasteur pipette and complete RPMI-10, pools of the eluted cells were placed in sterile 15 ml centrifuge tubes and spun at 250 g, 4°C for 10 min. For each tube, the cell pellet was resuspended in 6 ml complete RPMI-10. This volume was carefully layered over a 3 ml NycoPrep™ density gradient (Nycomed Pharma AS, Torshov, Norway) and centrifuged at 600 g, 20°C for 20 min. Mononuclear cells were removed from the interface ring using a Pasteur pipette, transferred to a sterile 15 ml centrifuge tube and spun down in 10 ml complete RPMI-10 at 400 g, 20°C for 15 min three times. The final pellet was resuspended in 1 ml of cold (4°C) Hanks balanced salt solution supplemented with 10% decomplemented FBS and 2 mM HEPES. Cells were quantified and viability assessed by trypan blue exclusion.

The final purification stage consisted of an immunomagnetic negative selection of DCs. The cell suspension obtained above was incubated under continuous agitation at 4°C for 1 hour, with primary rat anti-mouse monoclonal antibodies recognizing B and T lymphocytes, NK cells and monocytes/macrophages (1.5 μg/ml antibody per 1 × 10^6 ^cells). After incubation, cells were washed three times in Hanks centrifuging at 250 g, 4°C for 10 min. The pellet was resuspended in 1 ml cold Hanks in a sterile 15 ml centrifuge tube and incubated under continuous agitation at 4°C for 1 hour with a secondary sheep anti-rat IgG polyclonal antibody coupled to magnetic microspheres (Dynabeads^® ^M-450, Dynal Biotech Inc., Lake Success, U.S.A.) at a 7:1 sphere/target ratio. Non-dendritic magnetic-coated cells were removed by positive selection in three sequential depletions using a magnetic gadget (Dynal MPC^® ^Dynal Biotech Inc., Lake Success, U.S.A.) at 4°C for 6 min.

### Characterization of dendritic cells

DC purity was determined by flow cytometry and immunocytochemistry. For flow cytometry, 1 × 10^5 ^cells were suspended in PBS (1% FBS) and incubated with 10 μl primary monoclonal antibodies directly coupled to PE or FITC recognizing T and B lymphocytes, NK cells and monocytes/macrophages. DCs were characterized by an indirect method using primary monoclonal antibodies to CD11c and a secondary antibody, hamster anti-rat IgG1conjugated to FITC (clone MARG1-2, Serotec Ltd., Oxford, United Kingdom). The incubations were carried out in the dark at 4°C for 45 min, followed by 3 washes and centrifugation at 250 g, 4°C for 10 min. The cell pellet was resuspended in 500 μl PBS and the percentage of labeled cells determined in a flow cytometer (FACScan, Becton Dickinson, Franklin Lakes, U.S.A.). The control consisted of an antibody of irrelevant specificity conjugated to FITC.

For immunocytochemistry, 1 × 10^5 ^cells were suspended in PBS (1% FBS) and spun down at 50 g in a Cytospin (Shandon Inc., Pittsburg, U.S.A.). Sample slides were hydrated in PBS, fixed in fresh acetone for 5 min. and sequentially incubated for 90 min with primary rat monoclonal antibodies to CD11c and CD205, biotinylated goat anti-rat IgG (50 μg/ml) (Vector Laboratories, Burlingame, U.S.A.) for 45 min., and Vectastain^® ^Elite ABC kit (Vector Laboratories, Burlingame, U.S.A.) at 1:100, 30 min. Five-minute washes with PBS were done between incubations. The reactions were developed for 3 minutes in Vector^® ^NovaRed™ substrate. The slides were then washed and counterstained with methyl green. Omissions of the primary antibody and incubation with an antibody of irrelevant specificity at the same protein concentration were used as controls.

### Adoptive transfer of dendritic cells

DCs were isolated from the spleens of the following donor groups: a) Adult BALB/c mice infected during adulthood with *L. (L.). mexicana *(n = 4); b) Adult BALB/c mice infected during neonatal life with *L. (L.). mexicana *(n = 4); c) Healthy neonatal BALB/c mice (n = 4); d) Healthy adult BALB/c mice (n = 4). The infection control group consisted of neonatal BALB/c mice infected with 5 × 10^5 ^promastigotes of *L. (L) mexicana*.

DCs from the 4 experimental groups were adjusted to 1 × 10^3^, 1 × 10^5^, or 1 × 10^6 ^cells/ml in sterile PBS for intraperitoneal transfer to neonatal recipient BALB/c mice. Cells, at the mentioned concentrations, were injected in 20 μl volumes using a tuberculin syringe (29-gauge needle) connected to a stepper repetitive pipette (Tridak, Danbury, U.S.A.). After sixteen hours of adoptive transfer, neonatal recipient BALB/c mice were infected with 5 × 10^5 ^promastigotes of *L. (L) mexicana*.

Isolation of DCs and adoptive transfer experiments were done in duplicates.

### Statistical analysis

The results were expressed as mean ± standard error of the mean (SEM). Each experimental group consisted of 4–5 individuals. Comparisons between groups were made with Student t test and Welch t test for unpaired samples. Any value of p ≤ 0.05 was considered significant. All tests were performed using GraphPad InStat 3.02 (GraphPad Software, San Diego California USA, ).

## List of abbreviations

APCs: antigen-presenting cells

DCs: dendritic cells

TCR: T-cell receptor

## Competing interests

The author(s) declare that they have no competing interests.

## Authors' contributions

LVP carried out most of the experimental work and drafted the manuscript. JC developed the experimental design, carried out part of the experimental work and drafted the manuscript. NLD participated in the in experimental design and evaluated the progression of infection in the mice. FJT conceived the study, participated in the experimental design and coordinated the work. All authors read and approved the final manuscript.
